# Trigeminal TRPV1 regulates pneumococcal nose-to-brain invasion via IL-6/TNF-α signals

**DOI:** 10.1128/mbio.01335-25

**Published:** 2025-08-18

**Authors:** Hideki Sakatani, Masamitsu Kono, Masayoshi Hijiya, Yohei Morita, Tomoko Sumitomo, Tatsuya Inukai, Shigeki Nakamura, Yusuke Koizumi, Kenta Maruyama, Shizuya Saika, Masaaki Murakami, Muneki Hotomi

**Affiliations:** 1 Department of Otorhinolaryngology-Head and Neck Surgery, Wakayama Medical University 13145, Wakayama, Japan; 2 Department of Oral Microbiology, Tokushima University Graduate School of Biomedical Sciences 118112, Tokushima, Japan; 3 Department of Microbiology, Tokyo Medical University, Tokyo, Japan; 4 Department of Infection Prevention and Control, Wakayama Medical University 13145, Wakayama, Japan; 5 Department of Pharmacology, School of Medicine, Aichi Medical University 12703, Nagakute, Aichi, Japan; 6 Department of Ophthalmology, Wakayama Medical University 13145, Wakayama, Japan; 7 Division of Molecular Psychoimmunology, Hokkaido University, Institute for Genetic Medicine and Graduate School of Medicine 12810, Sapporo, Japan; 8 Group of Quantum immunology, Institute for Quantum Life Science, National Institute for Quantum and Radiological Science and Technology (QST) 13520 , Chiba, Japan; 9 Division of Molecular Neuroimmunology, Department of Homeostatic Regulation, National Institute for Physiological Sciences, National Institutes of Natural Sciences 34812, Okazaki, Aichi, Japan; The University of Mississippi Medical Center, Jackson, Mississippi, USA

**Keywords:** TRPV1, nose-to-brain infection, pneumococcus, host-pathogen interactions, invasive microorganisms

## Abstract

The nasal cavity acts as a gateway through which pathogens can invade the human body. *Streptococcus pneumoniae* can infect the central nervous system directly through the nasal cavity. However, the regulatory mechanisms that govern the direct nose-to-brain invasion of *S. pneumoniae* remain unclear. The nasal cavity is innervated by both olfactory and trigeminal nerves, which will modulate the defense mechanism of the olfactory epithelium in different ways. Transient receptor potential vanilloid 1 (TRPV1), which is expressed in both nerves, maintains the regeneration ability of the olfactory epithelium. In this study, we investigated the role of TRPV1 against the nose-to-brain invasion of *S. pneumoniae*. Six-week-old mice were treated with methimazole and/or resiniferatoxin to ablate TRPV1 on the olfactory and trigeminal nerves, respectively. Bacterial density, cytokines/chemokine induction, and histologic changes were investigated after intranasal infection with *S. pneumoniae*. Olfactory epithelium ablation by methimazole increased intracranial invasion and mortality without bacteremia. Trigeminal TRPV1 ablation suppressed mortality. Intracranial invasion of *S. pneumoniae* induced IL-6 in the olfactory bulb. Olfactory epithelium ablation was associated with significantly higher TNF-α compared to trigeminal TRPV1 ablation. Intracranial invasion was suppressed by intranasal administration of a TRPV1 stimulant. TRPV1 on the olfactory nerve worked against intracranial invasion, whereas TRPV1 on the trigeminal nerve enhanced lethality via excessive cytokine/chemokine production. The effect of intranasal TRPV1 stimulation was dominant on the olfactory nerve and could suppress central nervous system infection via the nose-to-brain route. The current finding can bring new strategies against infection targeting TRPV1.

**IMPORTANCE:**

This study updates the current clinical concept of the pathogenesis of pneumococcal meningitis and bacteremia. The findings provide new insight into how the non-hematogenous nose-to-brain route is controlled via transient receptor potential vanilloid 1 (TRPV1) of the olfactory and trigeminal nerve. A mouse model demonstrates the non-hematogenous invasion of *Streptococcus pneumoniae* from the nasal cavity directly into the cranium. TRPV1 on the olfactory nerve protected against intracranial invasion, whereas TRPV1 on the trigeminal nerve could induce a lethal course due to excessive inflammatory responses. Intranasal TRPV1 stimulation inhibited intracranial invasion and could control lethal central nervous system infection by *S. pneumoniae*. These findings suggest a novel preventive strategy targeting TRPV1 against invasive pneumococcal infections.

## INTRODUCTION

*Streptococcus pneumoniae* is a pathogen that frequently causes both invasive and non-invasive infections (1). Invasive pneumococcal infections such as pneumonia, meningitis, and sepsis remain a fatal threat, especially to compromised hosts such as children and elderly people (2–4). Even with the pneumococcal polysaccharide vaccines, invasive infections have not yet been eradicated, mainly due to the increase in non-vaccine strains (5, 6). In particular, meningitis remains fatal and causes severe complications (7, 8). Preventive strategies against the intracranial entry of *S. pneumoniae* remain a critical issue.

The most common route by which *S. pneumoniae* invades the central nervous system (CNS) is a hematogenous route from upper respiratory tract infection. However, recent studies have found a non-hematogenous route, i.e., direct intracranial invasion from the nose to the brain (9, 10). However, the regulatory mechanisms underlying the non-hematogenous nose-to-brain route remain unclear.

The nasal cavity contains the olfactory epithelium (OE), which is composed of olfactory receptor neurons (ORNs) (11). The stimulus is transmitted via axons of ORNs to the olfactory bulb (OB) in the cranium (12). OE has anatomically intracranial access as ORNs penetrate the pores of the lamina cribrosa and lead directly to OB. The unique constructions of the skull base and lamina cribrosa with pores are vulnerable to pathogens and can become an invasion route from the nose. However, the functions of OE in protecting CNS infections have not been investigated.

Immune responses are regulated by sensory nerves that detect environmental changes, including infections (13). OE is innervated by two sensory nerves: the olfactory nerve and the trigeminal nerve. Inputs from these sensory nerves can modulate the immune response to intranasal infection (13). Transient receptor potential (TRP) channels are sensitive to external stimuli and are expressed in a variety of cells throughout the body (14). Transient receptor potential vanilloid (TRPV) is one of the TRP subfamilies and is mainly found in sensory neurons for sensing temperature, pH, and pressure changes (15, 16). Notably, TRPV1 is abundantly present in the olfactory and trigeminal nerves (17). In the nasal cavity, TRPV1 was found in olfactory nerve fascicles to maintain an olfactory neuronal homeostasis, and in trigeminal nerve endings to facilitate the removal of foreign agents (18–20).

In this study, we hypothesized that TRPV1 functions differently in the olfactory and trigeminal nerves. We used a mouse model to demonstrate the nose-to-brain invasion of *S. pneumoniae* and to elucidate how TRPV1 on the two sensory nerves is involved in the infection. The aim of this study is to develop new treatment and prevention strategies against CNS infections caused by *S. pneumoniae*.

## RESULTS

### Distribution of TRPV1 in the nasal cavity

**Fig 1:**
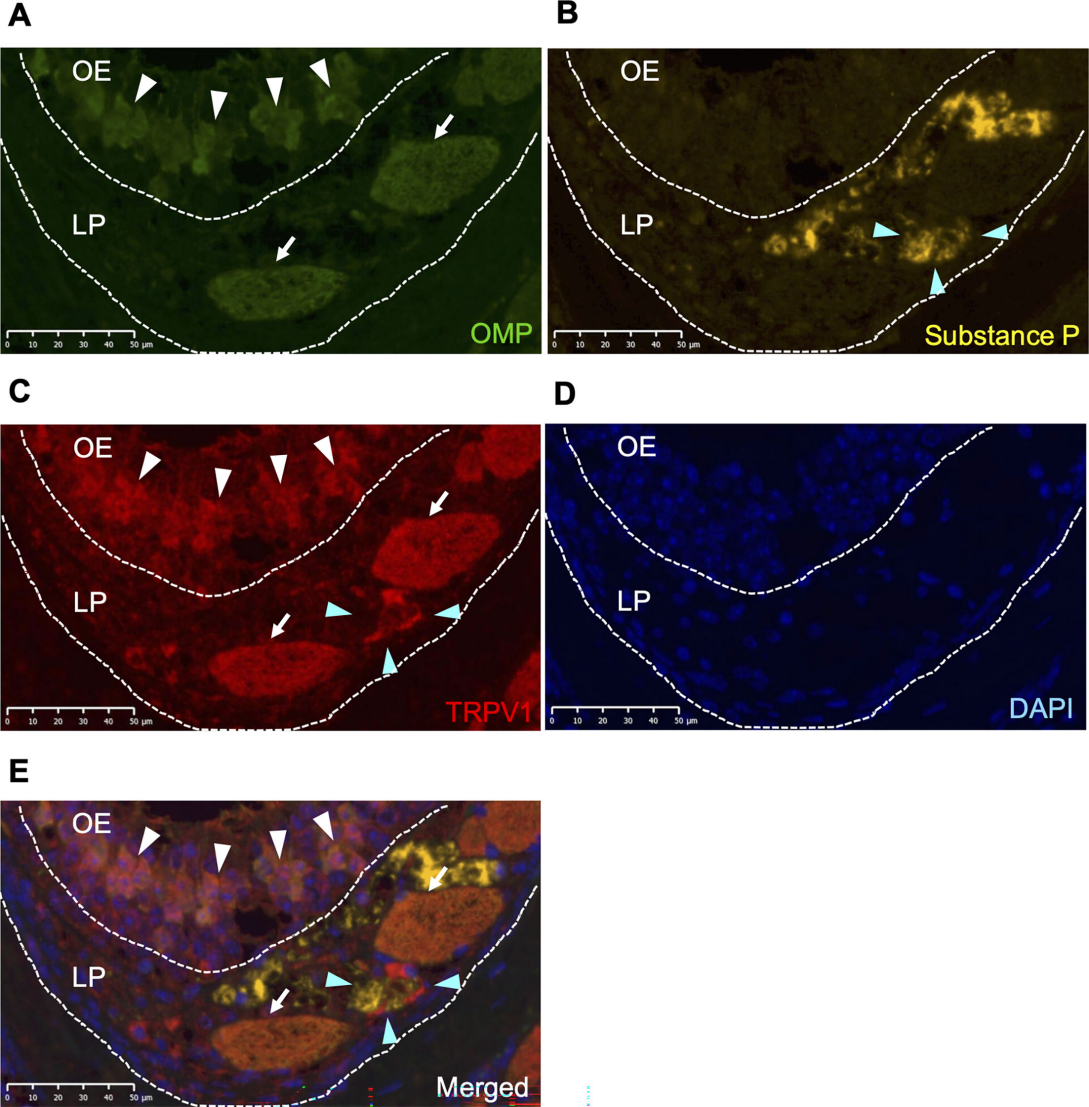
Distribution of the TRPV1 receptor in the nasal mucosa. Distribution of TRPV1 in WT mice was evaluated histologically in the nasal mucosa by immunofluorescent staining of (**A**) OMP (green), (**B**) substance P (yellow), (**C**) TRPV1 (red), (**D**) DAPI (blue), and (**E**) merged. White arrowheads, white arrows, and blue arrowheads represent ORN cell bodies, olfactory nerve fascicles, and trigeminal nerve fibers, respectively. Scale bar shown bottom right. LP, lamina propria; OE, olfactory epithelium; TRPV1, transient receptor potential vanilloid 1; OMP, olfactory marker protein; DAPI, 4',6-diamidino-2-phenylindole.

First, we performed an immunohistological examination to confirm the distribution of TRPV1 in the nasal cavity. Olfactory marker protein (OMP) is a specific antigen for ORNs (21, 22). Anti-OMP antibodies stained ORN cell bodies in OE (white arrowheads) and olfactory nerve fascicles in the lamina propria (LP) (white arrows) (Fig. 1A), as previously reported (18). Anti-substance P antibodies stain trigeminal nerve fibers (23). Anti-substance P-positive cells were located at different sites from the olfactory nerve fascicles in LP (blue arrowhead) (Fig. 1B and E). Anti-TRPV1 antibody and the merged image indicated that TRPV1 was present on ORN cell bodies in OE (white arrowheads) and in olfactory nerve fascicles in LP (white arrows), and around the trigeminal nerve fibers (blue arrowhead) (Fig. 1C and E). 4',6-Diamidino-2-phenylindole (DAPI) was used as a control (Fig. 1D). These findings showed that TRPV1 was expressed on both olfactory and trigeminal nerves in the nasal cavity.

### Experimental design and schematic diagram of the TRPV1 ablation model

**Fig 2:**
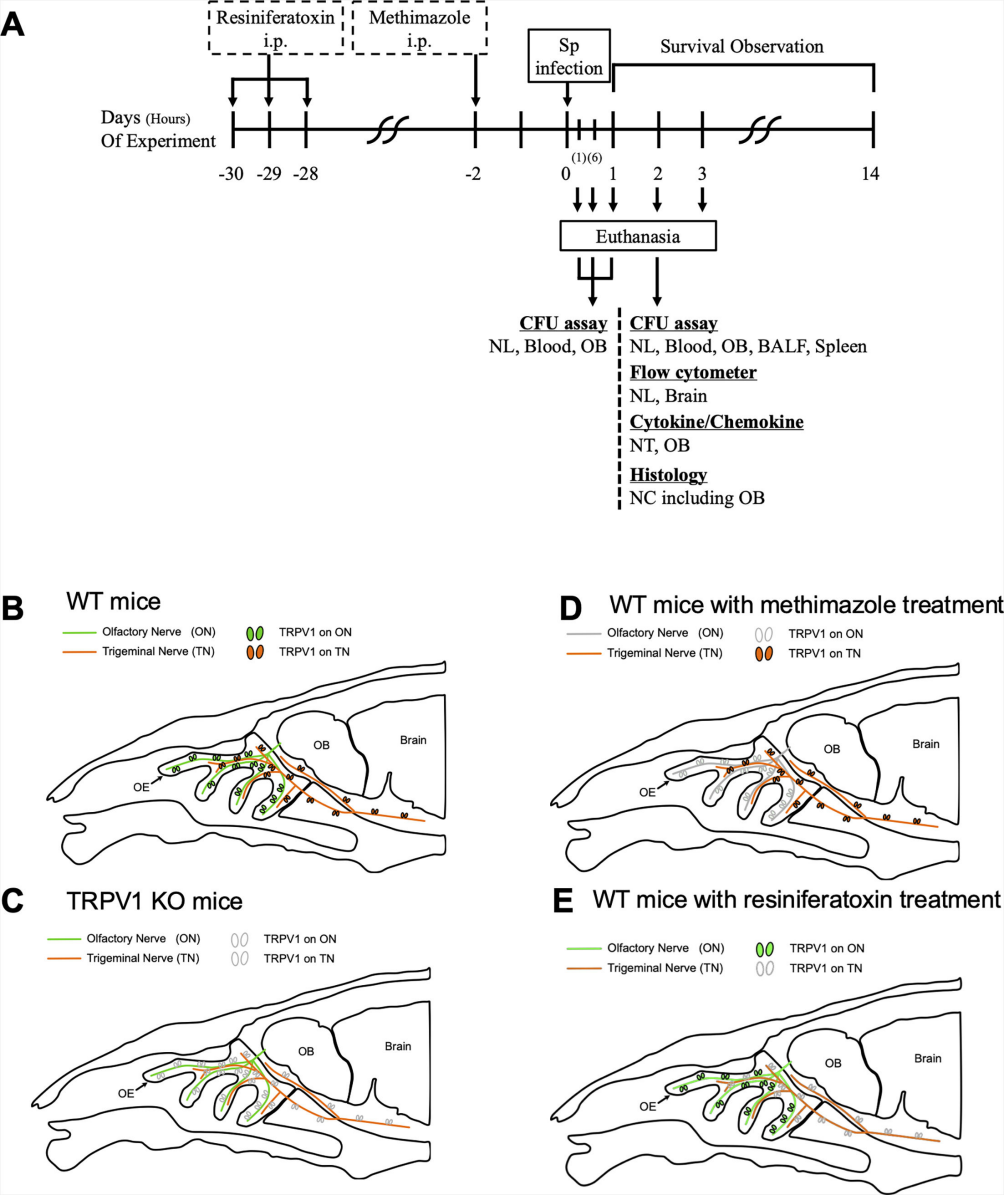
Experimental design and schematic diagram of the TRPV1 ablation model. (**A**) The experimental schedule of this study. Day 0 was defined as the day of *S. pneumoniae* infection. (**B**–**E**) Schematic illustration of the nasal cavity and cranium showing the distribution of TRPV1 on olfactory and trigeminal nerves. (**B**) WT mice. The olfactory nerve, including ORNs, extends from the nasal cavity to the cranium. The trigeminal nerve is also innervated in the nasal cavity and cranium. TRPV1 is present on both nerves. (**C**) TRPV1 KO mice. TRPV1 in the nasal cavity and cranium is defective. (**D**) WT mice with MZ treatment. ORNs and TRPV1 on them are depleted. (**E**) WT mice with RTX treatment. TRPV1 on the trigeminal nerve is ablated.

The experimental schedule was shown (Fig. 2A). Day 0 was defined as the day when mice were infected intranasally with 7,000 CFUs of *S. pneumoniae* under anesthesia with isoflurane. The anatomical distribution of TRPV1 in the nasal cavity of wild-type mice was shown (Fig. 2B). TRPV1 on olfactory and trigeminal nerves was lost in TRPV1 KO mice (Fig. 2C). Methimazole (MZ) was used to selectively damage ORNs, including TRPV1 (Fig. 2D) (18). Resiniferatoxin (RTX) is one of the agonists of TRPV1, but it causes long-lasting desensitization and functional ablation of TRPV1 mainly on nociceptive nerves due to its strong activity (24). The nociceptive nerve innervating the nasal cavity is the trigeminal nerve, so we used RTX-pretreated mice as a model of TRPV1 dysfunction in the trigeminal nerve (Fig. 2E). To confirm the effect of MZ and RTX, the expression of TRPV1 was examined by immunofluorescence staining after MZ or RTX treatment (Fig. S1). OE and LP were thinner in MZ-treated mice than in untreated mice in Fig. 1 (Fig. S1A). There were OMP- and TRPV1-co-positive cells only in olfactory nerve fascicles (white arrows), while TRPV1 was still present around trigeminal fibers (blue arrowheads) (Fig. S1A–1E). ORNs, including TRPV1, were impaired by MZ. In contrast, TRPV1 was found in ORN cell bodies (white arrowheads) and olfactory nerve fascicles (white arrows), but not around trigeminal fibers (blue arrowheads) in RTX-treated mice (Fig. S1I). Expression of OMP and substance P resembled untreated mice in Fig. 1. DAPI was used as a control (Fig. S1EJ). These findings were consistent with the schematic illustration of MZ or RTX-treated mice in Fig. 2D and E.

### Intracranial invasion of a virulent *S. pneumoniae* strain increased in TRPV1 KO mice

**Fig 3:**
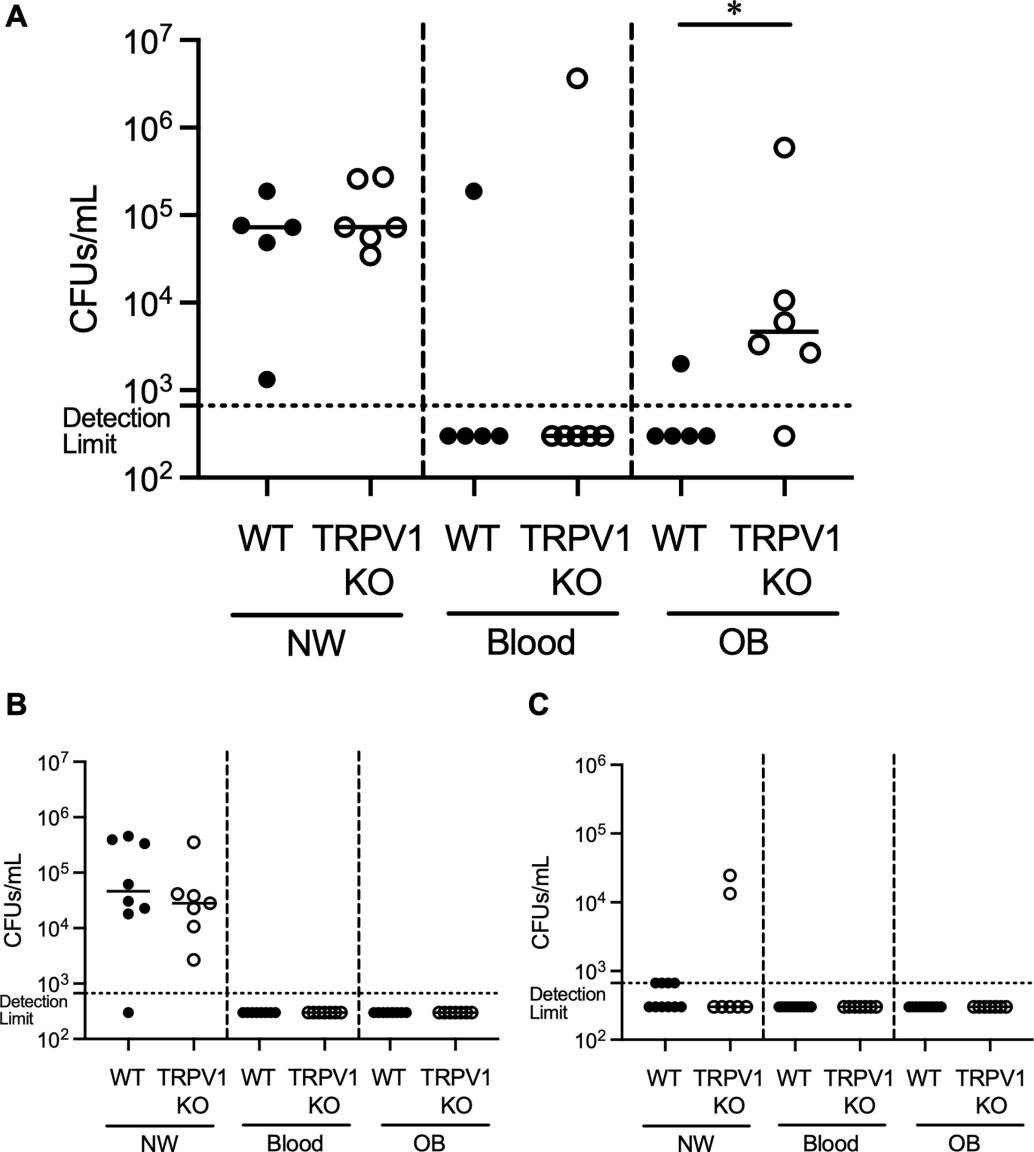
Intracranial invasion of a virulent *S. pneumoniae* strain increased in TRPV1KO mice. WT and TRPV1 KO mice were used. Each mouse was infected intranasally with 7,000 CFUs/mouse of *S. pneumoniae*. They were euthanized 48 hours after infection, and the bacterial amounts in nasal lavage (NW), blood (blood), and olfactory bulb (OB) were counted. (**A**) Mice infected with 6A. WT mice (*n* = 5) and TRPV1 KO mice (*n* = 6), respectively. (**B**) Mice infected with 19F. WT mice (*n* = 8) and TRPV1 KO mice (*n* = 7), respectively. (**C**) Mice infected with MNZ11. WT mice (*n* = 10) and TRPV1 KO mice (*n* = 7), respectively. One dot represents one mouse. The averages were indicated by the bar. Statistical comparisons were performed using the Mann-Whitney U test. *P < 0.05.

We evaluated the influence of TRPV1 on the dynamics of *S. pneumoniae* nasal infection between TRPV1 KO and WT mice. The experimental schedule is shown in Fig. 2A; the samples were obtained on day 2. Infected intranasally with 6A, a virulent strain (25), higher bacterial loads were detected in OB of TRPV1 KO mice. There were no significant differences in nasal lavage or blood (Fig. 3A). Both serotypes 19F and a non-encapsulated pneumococcal strain of MNZ11 are considered less virulent than 6A (26, 27). In 19F and MNZ11-infected mice, no bacteria were observed in blood or OB in either TRPV1 KO or WT mice (Fig. 3B and C). Thus, the influence of TRPV1 against intracranial invasion was prominent with the virulent strain.

### Ablation of TRPV1 on the olfactory nerve increased intracranial invasion of *S. pneumoniae* serotype 6A and lethality, whereas ablation of TRPV1 on the trigeminal nerve suppressed lethality

**Fig 4:**
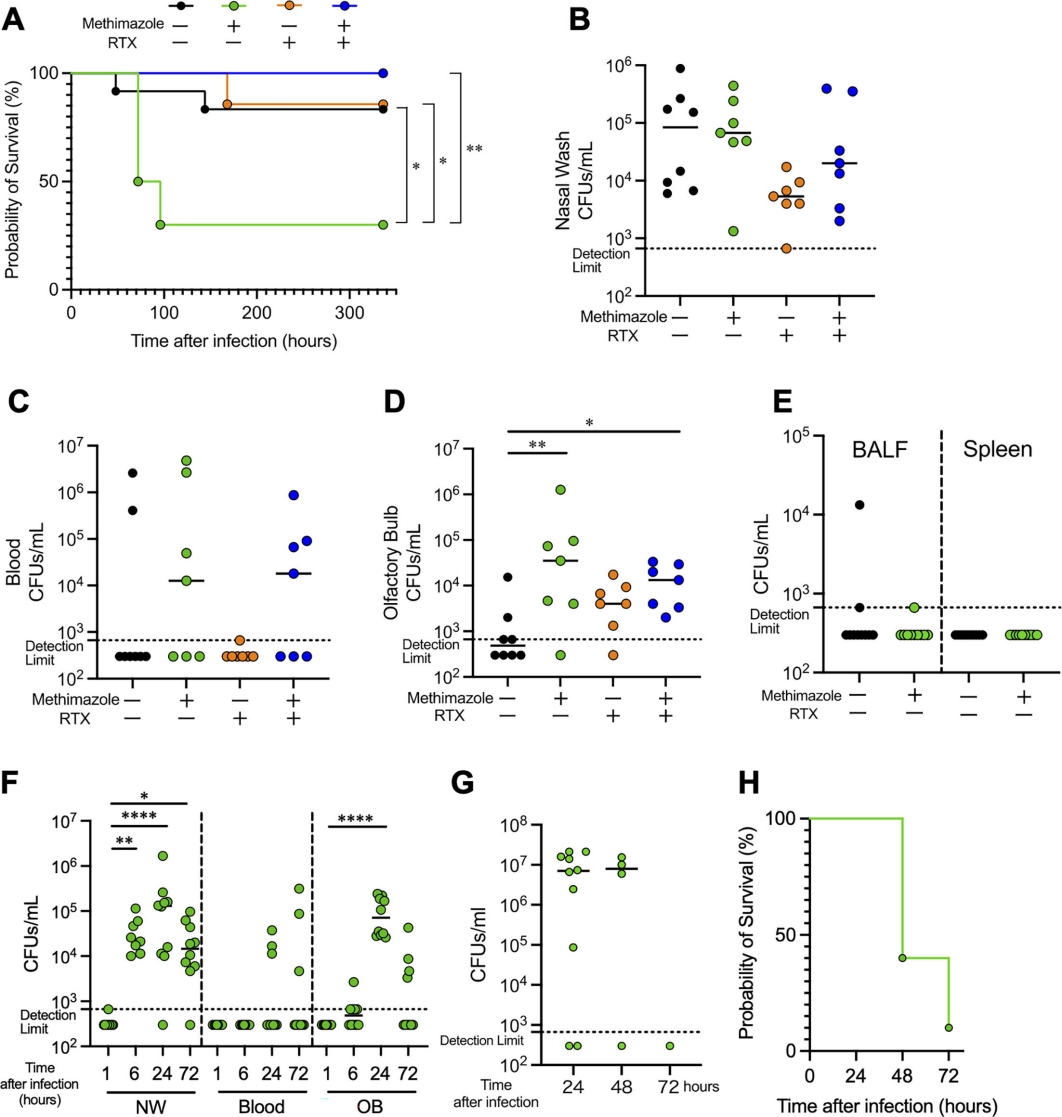
Ablation of TRPV1 on the olfactory nerve increased intracranial invasion of *S. pneumoniae* serotype 6A and lethality, whereas ablation of TRPV1 on the trigeminal nerve suppressed lethality. WT mice were pretreated with MZ and/or RTX according to the procedures shown in Fig. 2A, followed by intranasal infection with 6A at 7,000 CFUs/mouse. (**A**) Survival curves after infection. (**B**–**E**) The bacterial amount in the following tissues at 48 hours post-infection. (**B**) Nasal lavage. (**C**) Blood. (**D**) Olfactory bulb suspension. (**E**) Bronchoalveolar lavage fluid (BALF) and spleen. (**F**) Nasal lavage, blood, and olfactory bulb suspension of MZ-mice at 1, 6, 24, and 72 hours post-infection. Black dots represent mice without pretreatment of MZ or RTX (control mice). *n* = 10 (**A**), 8 (**B–D**), and 10 (**E**). Green dots represent mice pretreated with MZ (MZ-mice). *n* = 12 (**A**), 7 (**B–D**), and 10 (**E, F**). Orange dots represent mice pretreated with RTX (RTX-mice). *n* = 9 (**A**) and 7 (**B–D**). Blue dots represent mice pretreated with MZ and RTX (MZ-RTX-mice). *n* = 7 (**A–E**). (**G**–**H**) Intracranial infection model in MZ-mice. (**G**) Bacterial load in blood after intracranial infection. Blood was collected from surviving mice at 24 (*n* = 10), 48 (*n* = 4), and 72 hours (*n* = 1) post-infection. (**H**) Survival curves following intracranial infection. NW, nasal lavage; OB, olfactory bulb suspension. Each dot represents one mouse. Statistical comparisons to the control were performed using the Log rank test (**A**), Kruskal-Wallis test with Dunn’s multiple-comparison test (**B–D, F**), and Mann-Whitney U test (**D**). *P < 0.05, **P < 0.01, and ****P < 0.001.

We investigated survival after intranasal infection with 6A to determine the differential TRPV1 function of the olfactory nerve or the trigeminal nerve. Mice treated with MZ (MZ-mice) showed significantly lower survival rates than mice without either MZ or RTX treatment (control mice), mice treated with RTX (RTX-mice), and mice treated with both MZ and RTX (MZ-RTX-mice). The additional RTX treatment to MZ treatment improved the survival rate (Fig. 4A).

To investigate changes in the *S. pneumoniae* dynamics following MZ and/or RTX treatment, bacterial amount in nasal lavage, blood, and OB was quantified at 48 hours after infection with 6A, a time point at which most of the mice survived. The colonization density of 6A was not different among groups (Fig. 4B). All groups exhibited few bacteremia (Fig. 4C). In OB, MZ-mice and MZ-RTX-mice showed significantly higher density than control mice (Fig. 4D). To exclude the possibility that the invasion into OB could have resulted from bacteremia following lower respiratory tract infection or from the spleen during the eclipse phase, we evaluated both bronchoalveolar lavage fluid (BALF) and spleen at 48 hours post-infection (28). 6A was not detected in either BALF or spleen in both control and MZ-mice, except for one case with BALF (Fig. 4E). The bacterial amount of lung was consistent with a previous report (29). In contrast, the absence of bacteria in the spleen will reflect a different route of infection. We further investigated the kinetics of nose-to-brain invasion of *S. pneumoniae* in MZ-mice. 6A in nasal lavage and OB peaked at 24 hours post-infection and declined thereafter (Fig. 4F). 6A in blood remained low over time. The bacterial loads in any tissues were not different at 24 hours compared to 48 hours post-infection (Fig. 4B through D and F). Based on these results, we hypothesized that intranasally infected *S. pneumoniae* directly caused intracranial infection and then developed bacteremia, leading to death. To prove this hypothesis, 7,000 CFUs of 6A were infected intracranially in MZ-mice, reflecting the average bacterial load in OB of MZ-mice in Fig. 4D. Bacteremia developed in most mice after intracranial infection: 8 out of 10 survivors at 24 hours and 3 out of 4 survivors at 48 hours (Fig. 4G). One mouse survived without bacteremia at 72 hours (Fig. 4H). These findings suggest that the bacteria presence in the blood will have resulted from the brain, across the blood-brain barrier, into the systemic circulation. TRPV1 dysfunction on the olfactory nerve exacerbated lethality by facilitating CNS invasion, whereas TRPV1 dysfunction on the trigeminal nerve increased intracranial invasion but attenuated lethality.

### *S. pneumoniae* serotype 6A invaded OB along fibers of the olfactory nerve

**Fig 5:**
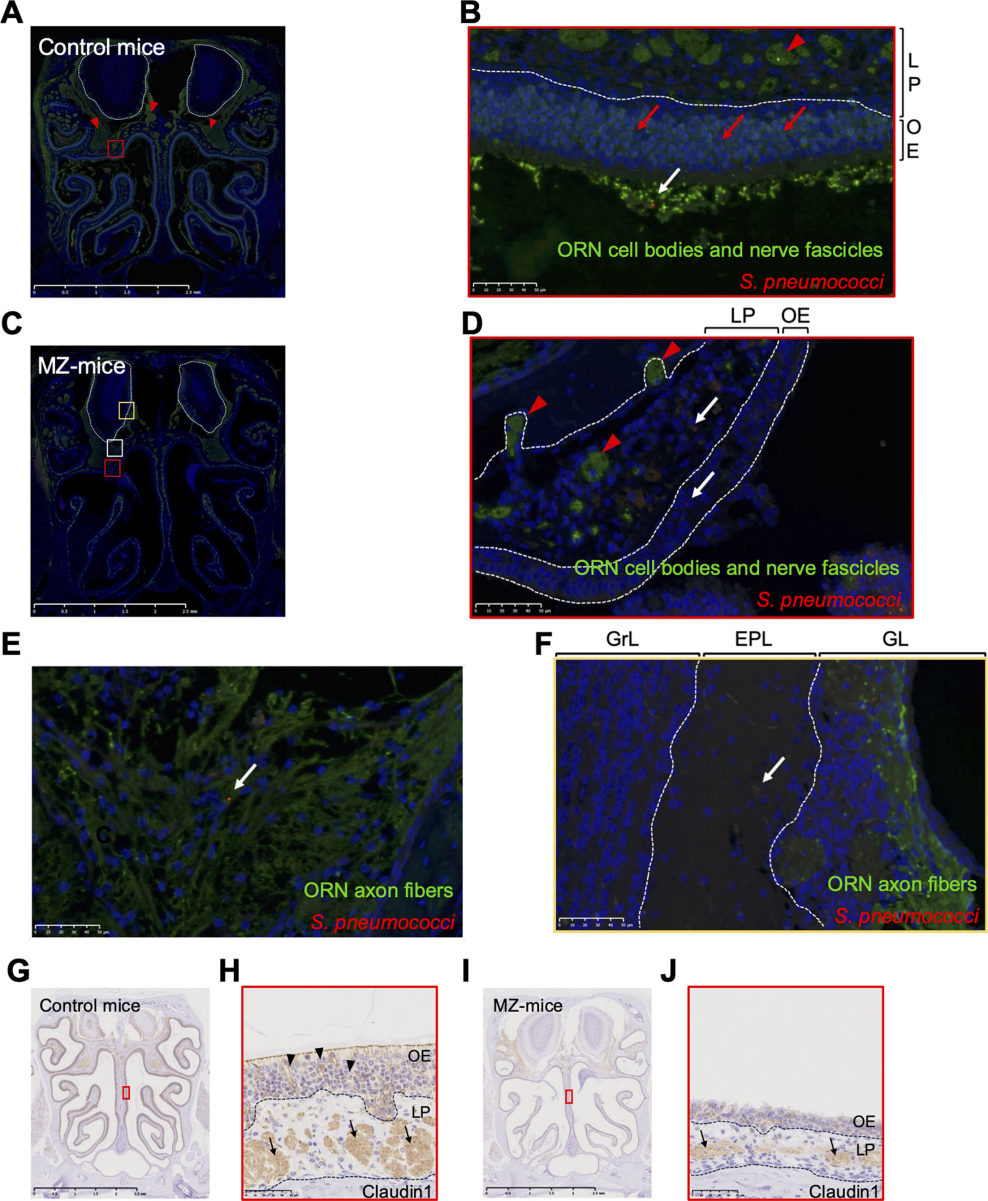
*S. pneumoniae* serotype 6A invaded OB along fibers of the olfactory nerve. Representative immunofluorescence histological images of coronal sections of the nose at 48 hours post-6A infection. (**A**–**B**) Control mice. (**C**–**F**) MZ-mice. ORNs and axon fibers were labeled green. 6A was labeled in red. Cell nuclei were labeled in blue with DAPI. Red arrowheads, red arrows, and white arrows represent axon fibers of ORNs, cell bodies of ORNs, and 6A, respectively. A scale bar is shown in the lower right corner of each image. (**A**) Low magnification image. The areas outlined by white dashed lines represent the olfactory bulb. (**B**) Magnified image of the red boxed area in A. (**C**) Low magnification image. The areas outlined by white dashed lines represent the olfactory bulb. (**D**) Magnified image of the red boxed area in C. (**E**) Magnified image of the white boxed area in C. The area represents the intracranial space between the OE and the olfactory bulb. (**F**) Magnified image of the yellow boxed area in C. The area represents the inside of the olfactory bulb. (**G**–**J**) Representative immunohistochemical images of claudin-1 in OE. (**G**, **I**) Low magnification images from control (**G**) and MZ-treated (**I**) mice. (**H**, **J**) High magnification of the red boxed areas in **G** and **I**, respectively. Black arrowheads indicate the intercellular space between ORN cell bodies, and black arrows indicate olfactory nerve fascicles in LP. OE, olfactory epithelium; LP, lamina propria; GrL, granule cell layer; EPL, external plexiform layer; GL, glomerular layer.

We performed immunofluorescent staining to elucidate the invasion route from the nose to the OB, evaluating the nasal coronal sections at 48 hours post-infection. In control mice, OMP-positive cells were abundant in ORN cell bodies in OE (red arrow) and in the olfactory nerve fascicles (red arrowhead) in LP and the cranium (Fig. 5A and B). 6A was found only on the surface of OE and did not invade into OE or LP (Fig. 5B). In MZ-mice, the OMP-positive cells decreased, reflecting ORN damages (Fig. 5C). In contrast, 6A was found inside OE and LP (Fig. 5D), on olfactory nerve fascicles toward OB in the cranium (Fig. 5E), and in the external plexiform layer (EPL) inside OB (Fig. 5F) (white arrows). To assess the impact of MZ on tight junctions, expression of claudin-1, a tight junction protein on ORNs, was examined by immunostaining (30). In control mice, claudin-1 was expressed in the space between ORN cell bodies in OE (black arrowheads) and in ORN nerve fascicles in LP (black arrows) (Fig. 5G and H). However, in MZ-treated mice, the expression of claudin-1 was reduced in both OE and LP (Fig. 5I and J). 6A migrated into OE and invaded OB along the remaining ORN fibers under ORN and tight junction damages.

### Inflammatory responses in the nasal cavity and the OB

**Fig 6:**
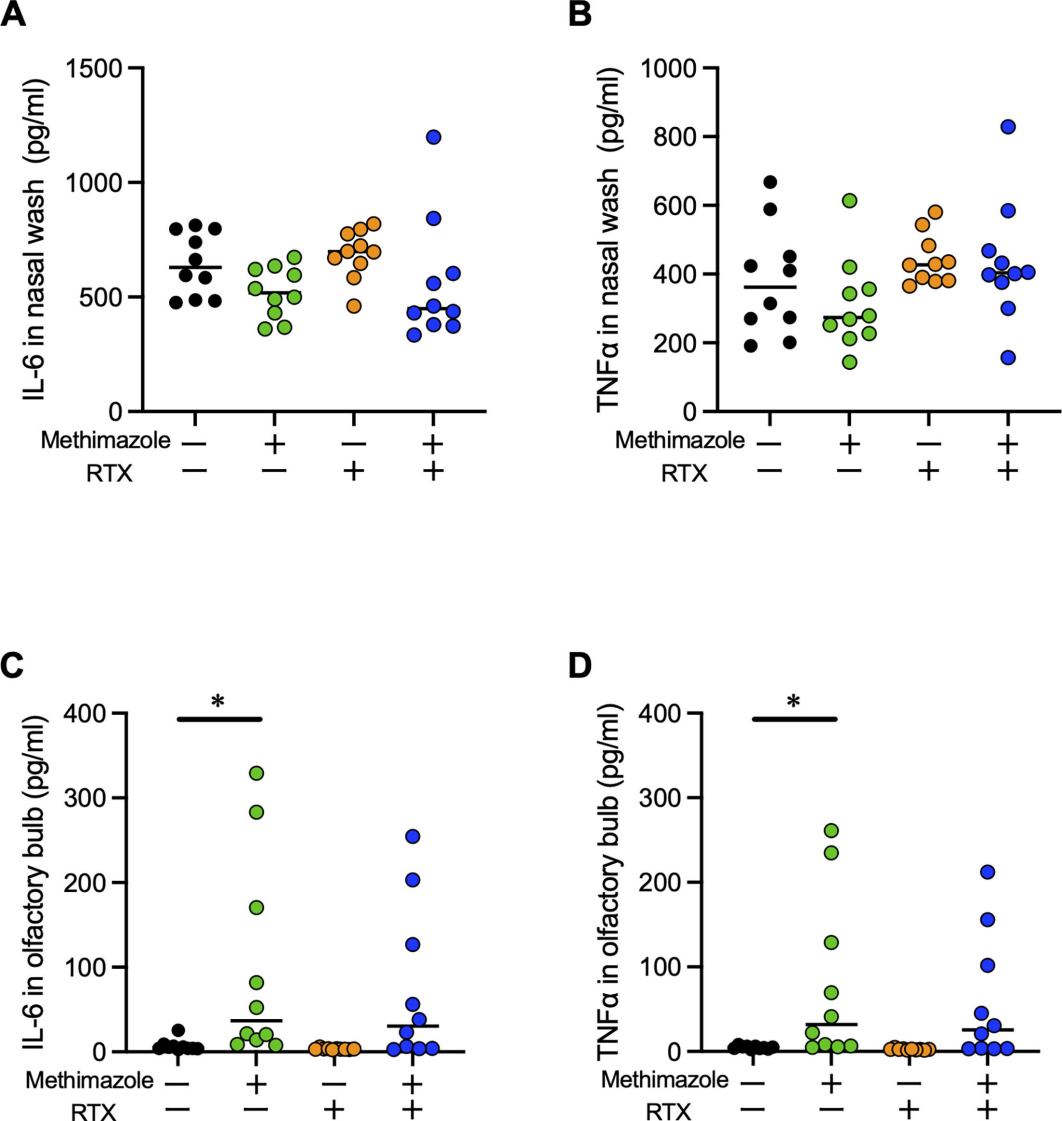
Inflammatory responses in the nasal cavity and OB by ELISA. WT mice were pretreated with MZ and/or RTX as shown in Fig. 2A, then euthanized at 48 hours post-infection. (**A**, **C**) IL-6 levels in nasal lavage (**A**) and OB (**C**). (**C**, **D**) TNF-α levels in nasal lavage (**B**) and OB (**D**). Black dots represent control mice. Green dots represent MZ-mice. Orange dots represent RTX-mice. Blue dots represent MZ-RTX-mice. *n* = 10 for all groups. Statistical comparisons were performed using the Kruskal-Wallis test with Dunn’s multiple-comparison test. *P < 0.05.

When comparing MZ-mice with MZ-RTX-mice in the survival rate (Fig. 4A) and the bacterial amount of OB (Fig. 4D), the intracranial invasion and the lethality were not parallel. To investigate this discrepancy, we quantified IL-6 and TNF-α as main cytokines against bacterial infection by enzyme-linked immunosorbent assay (ELISA). Although neither cytokine showed significant differences among all groups in nasal tissue (Fig. 6A and B), both IL-6 and TNF-α levels were significantly elevated in OB of MZ-mice compared to control mice (Fig. 6C and D). In addition, we investigated other inflammatory cytokines/chemokines, neuropeptides, and growth factors via quantitative real-time PCR assay. Neutrophils are one of the main innate immune cells opposing intranasal *S. pneumoniae* infection, and CXCL1 induces neutrophil chemotaxis (26). The levels of *Cxcl1* in OB of MZ-mice and MZ-RTX-mice showed predominantly higher levels compared with control mice (Fig. S2A and B). The release of cGRP, a neuropeptide from C-fibers, is facilitated by TRPV1 activation (31). Only MZ-mice showed predominantly higher *cgrp* levels in OB (Fig. S2C and D). The levels of *Il-6* and *Tnf-α* in OB were also significantly higher in MZ-mice (Fig. S2FH), but not different in nasal tissue (Fig. S2EG). The results were consistent with ELISA. Since TRPV1 promotes tissue regeneration of ORNs, we also investigated the levels of nerve growth factor (NGF) (32, 33). The *Ngf* levels did not differ among groups (Fig. S2I and J). The numbers of neutrophils and macrophages in nasal lavage were not different among groups (Fig. S2K and L). To assess the impact of infection route on inflammatory cell infiltration, nasal lavage and OB homogenate from MZ-mice were analyzed after intraperitoneal or intranasal *6A* infection. There were no statistically significant differences in neutrophil and macrophage levels between the two infection routes (Fig. S2M and N). These results suggested that IL-6 and TNF-α levels were consistent with the lethality observed in MZ-mice, whereas the association with immune cell infiltration was unclear.

### Increased IL-6/TNF-α levels were accompanied by intracranial NFκB/STAT3 activation

**Fig 7:**
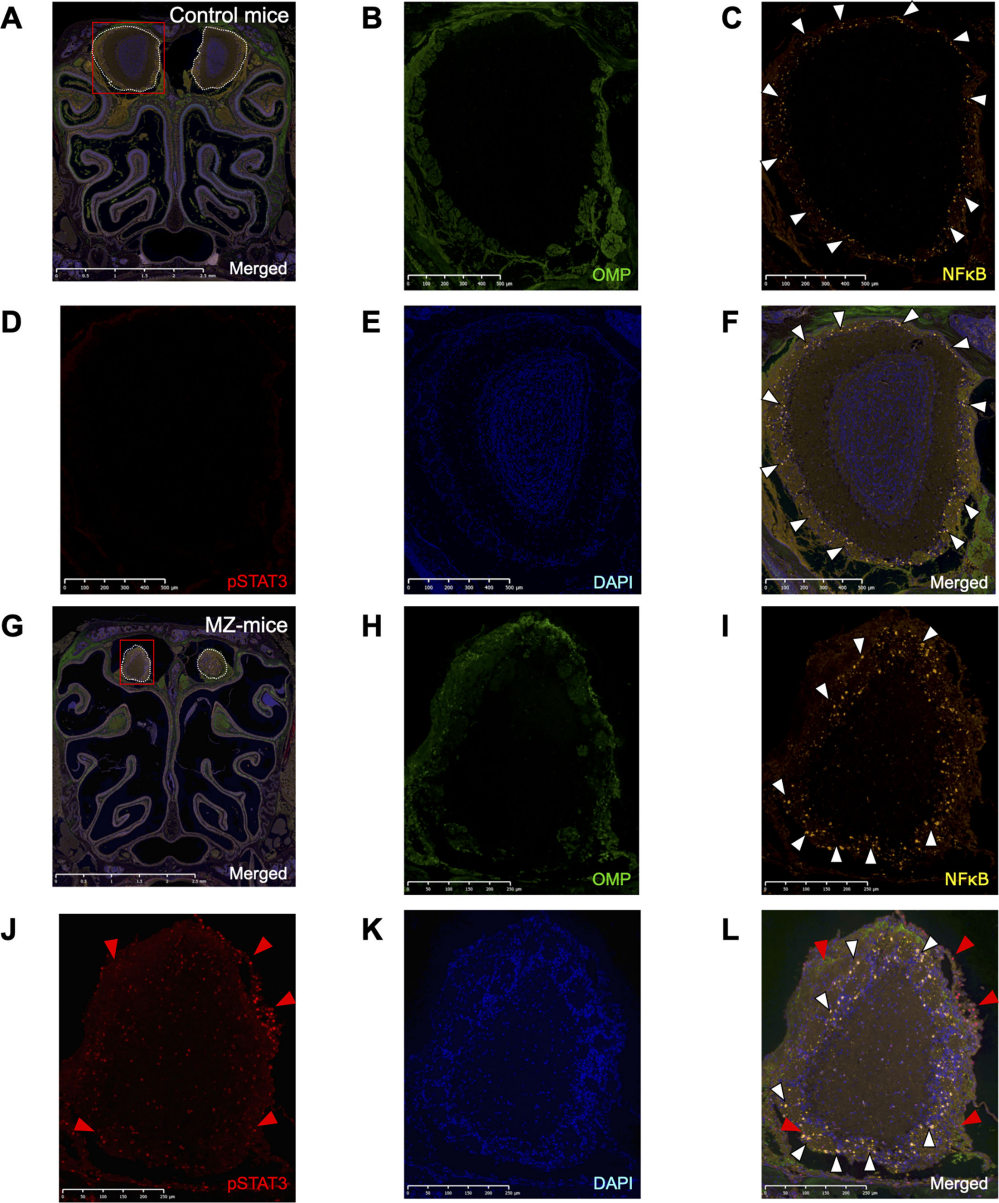
Increased IL-6/TNF-α levels were accompanied by intracranial NFκB/STAT3 activation. Representative immunofluorescence histological images of the OB at 48 hours post-6A infection. (**A**–**F**) Control mice. (**G**–**L**) MZ-mice. ORN cell bodies and axon fibers were labeled green. NFκB was labeled in yellow. pSTAT3 was labeled in red. Cell nuclei were labeled in blue with DAPI. White arrowheads and red arrowheads represent NFκB and pSTAT3 activation around the OB, respectively. A scale bar is shown in the lower right corner of each image. (**A**, **G**) Low magnification image. The areas outlined by white dashed lines represent the olfactory bulb. (**B**–**F**, **H**–**L**) Magnified image of the red boxed area in **A**, **G**. (**B**, **H**) OMP. (**C**, **I**) NFκB. (**D**, **J**) pSTAT3. (**E**, **K**) DAPI. (**F**, **L**) Merged.

To elucidate why increased IL-6 and TNF-α levels correlated with lethality, the distribution of NFκB and STAT3 activation was evaluated by fluorescence immunostaining at 48 hours post-infection. In control mice, there was only weak NFκB activation (white arrowheads) and no detectable pSTAT3 activation in OB (Fig. 7A through F). In contrast, MZ-mice showed pSTAT3 activation (red arrows) along with NFκB activation (white arrowheads) (Fig. 7G through L). The elevated IL-6 and TNFα levels were accompanied by NFκB and STAT3 activation.

### TRPV1 stimulation inhibited intracranial invasion caused by OE damage

**Fig 8:**
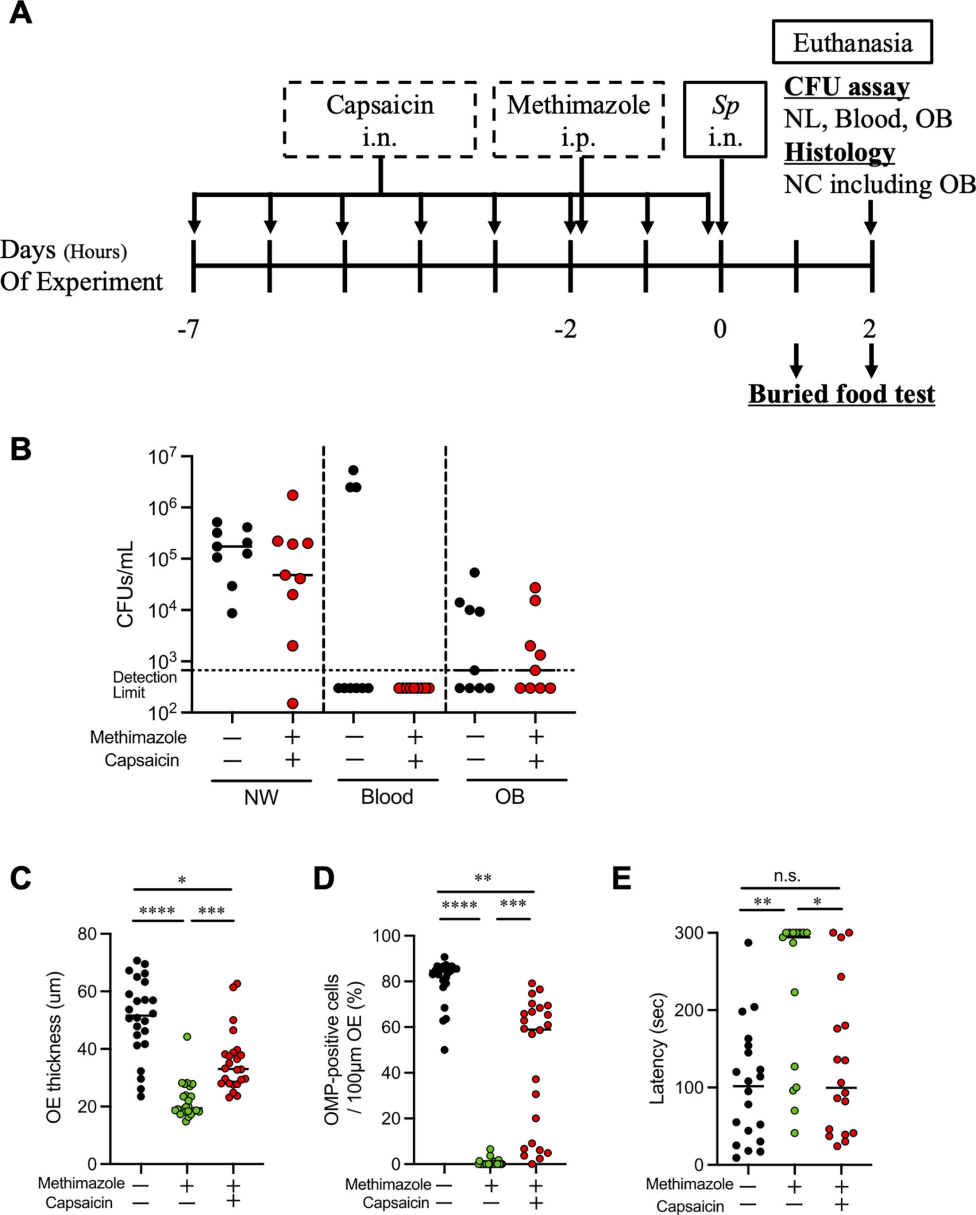
TRPV1 stimulation inhibited intracranial invasion caused by OE damage. (**A**) Experimental schedule including CAP and MZ pre-treatment. Day 0 was defined as 6A infection. (**B**) Bacterial amount in nasal lavage (NW), blood, and olfactory bulb suspension (OB) at 48 hours post-infection. Mice without either CAP nor MZ pretreatment were compared with both CAP and MZ pretreatment. (**C**–**D**) Histological analysis using images of nasal coronal sections at 48 hours post-infection. (**C**) OE thickness. (**D**) OMP-positive cell ratio. (**E**) Buried food test at 24 and 48 hours post-infection. The extension of latency indicated poor olfaction. Black dots represent mice without pretreatment of CAP nor MZ (control). *n* = 9 (**B**), 4 (**C, D**), and 10 (**E**). Red dots represent mice pretreated with CAP and MZ (CAP-MZ-mice). *n* = 9 (**B**), 4 (**C, D**), and 9 (**E**). Green dots represent mice pretreated with MZ (MZ-mice). *n* = 4 (**C, D**) and 8 (**E**). Statistical comparisons were performed using Mann–Whitney U test (**B**) and Kruskal-Wallis test with Dunn’s multiple-comparison test (**C–E**). *P < 0.05, **P < 0.01, ***P < 0.005, and ****P < 0.001.

Capsaicin (CAP) is a TRPV1 agonist and a common chemical component of chili peppers (34). We investigated whether intranasal TRPV1 stimulation could suppress 6A nose-to-brain invasion according to the experimental schedule (Fig. 8A). In this experiment, mice pre-treated with CAP were defined as CAP-mice, and those pre-treated with both capsaicin and MZ were defined as CAP-MZ-mice, respectively. CAP-MZ-mice showed no significant differences in the bacterial amount in nasal lavage, blood, and OB compared with control mice (Fig. 8B). The bacterial amount in OB was significantly lower in CAP-MZ-mice than in MZ-mice in Fig. 4D (*P* < 0.05).

Compared with control mice, MZ-mice were shown to have significantly thinner OE (Fig. 8C), lower OMP-positive cell rates (Fig. 8D), and longer latency for the buried food test (Fig. 8E). CAP-MZ-mice also exhibited significantly thinner OE and lower OMP-positive cell rates than control mice, while both OE thickness and OMP-positive cell rate were higher than in MZ-mice (Fig. 8C and D). There was no statistical difference in latency between CAP-MZ-mice and control mice (Fig. 8E). These findings indicated that CAP suppressed MZ-induced olfactory dysfunction and intracranial invasion of 6A, followed by ORN damage.

## DISCUSSION

*S. pneumoniae* differed in their ability to invade the intracranial space according to serotype (10, 35). Although all three strains used in this study can cause meningitis in humans, serotype 6A was suitable for the model for studying TRPV1-mediated nose-to-brain invasion (27).

The first step of *S. pneumoniae* infection is entry into the nasal cavity. *S. pneumoniae* initially colonizes the nasal mucosa and then enters the lower respiratory tract, the bloodstream, or the intracranial space to cause invasive infections (36). ORNs in OE can act as a barrier against CNS invasion (37). Our study showed that intracranial invasion of *S. pneumoniae* was mainly facilitated by OE damage, including TRPV1 on ORNs, and potentially by disruption of tight junctions on ORNs. Furthermore, the intracranial invasion of *S. pneumoniae,* followed by OE damage, was not accompanied by blood, spleen, or lung invasion. And the similar time dependence of bacterial loads in nasal lavage and OB suggested that 6A rapidly entered the cranium via the non-haematogenic route. In a comparable model using pups, bacteremia did not develop until 4 days after intranasal infection (38). The nose-to-brain route will likely develop meningitis more quickly compared to the hematogenic route. Additionally, *S. pneumoniae* was detected in the bloodstream as early as 24 hours after intracranial infection, consistent with a previous finding (39). The non-haematogenic nose-to-brain invasion followed by bacteremia will underlie the lethality in this model.

Nose-to-brain invasion routes for pathogens include transport within ORN axons and olfactory nerve ensheathing cells (OECs), or along extracellular perineural spaces of ORNs (40–42). Methimazole induces detachment of the supporting cells and death of ORNs but does not show toxicity to OECs (43, 44). In this study, ORN fibers remained in LP and the cranium, although ORN cell bodies in OE were lost after methimazole treatment (45). The ORN axon fiber with a diameter of 0.2 µm is insufficient for internalizing bacteria, so *S. pneumoniae* will intrude intracranially along ORN axons (46). Since active transport pathways are unlikely to be active in residual axons after methimazole treatment, the perineural spaces along the axons would be involved. Pericellular sialic acid facilitates transport in the nervous system (47). Intact ORN cell bodies worked against *S. pneumoniae* invasion, but the axons with sialic acid might be utilized as an invasion pathway under ORN damage.

The nasal cavity is exposed to a variety of external stimuli. OE is repeatedly damaged and regenerated throughout life (48). Common upper respiratory tract infections can also trigger OE damage (37, 49). These facts suggest that undetected cycles of OE damage and repair transiently modulate the host’s vulnerability to CNS invasion. Contributing to the ORN regenerative process (18), TRPV1 dysfunction should prolong vulnerability to CNS invasion by impairing epithelial repair mechanisms.

**Fig 9:**
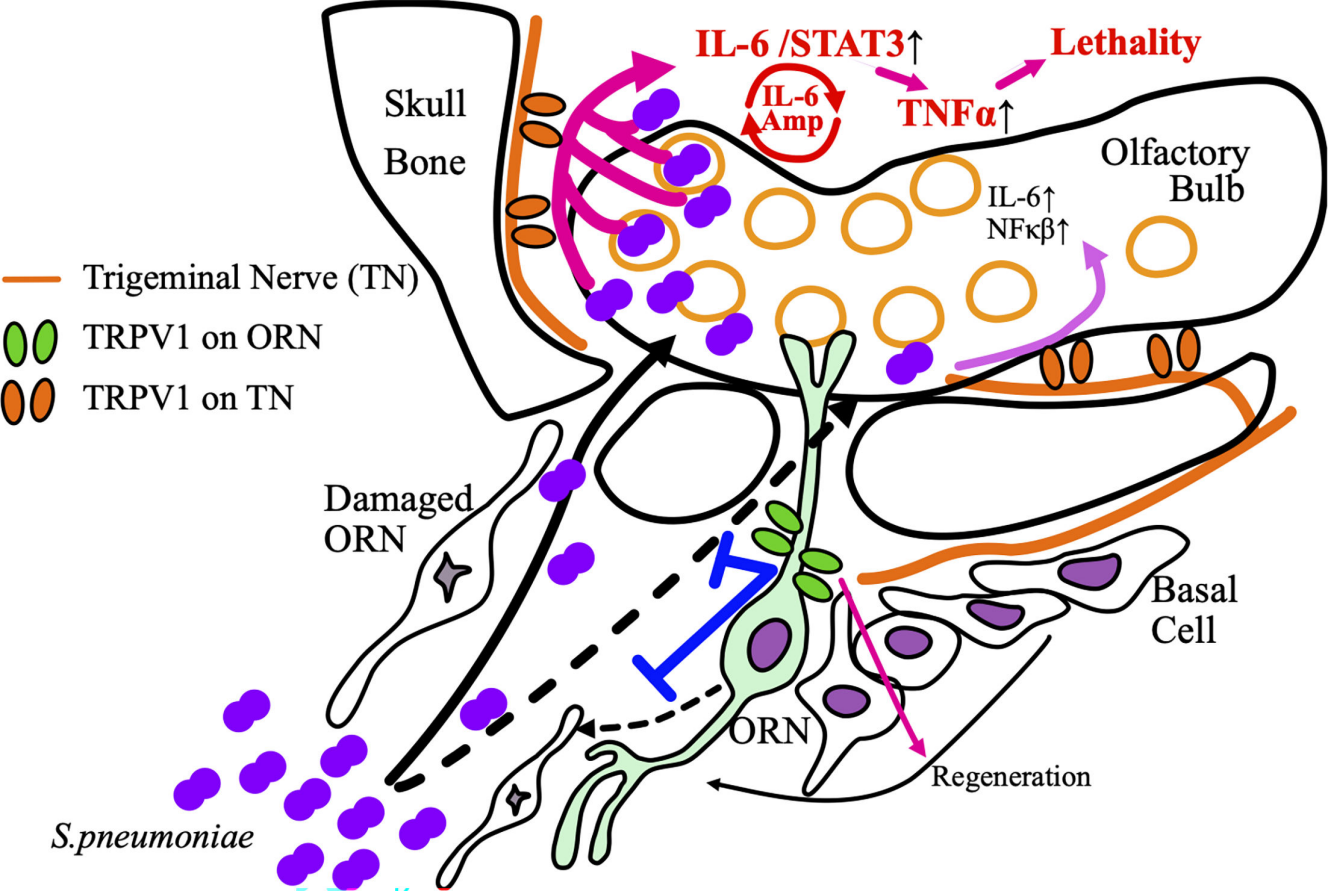
Schematic illustration of the dynamics of *S. pneumoniae* in the OE and the role of TRPV1. OE containing ORNs and OB are fringed by the cranial bone. The skull base has small pores through which ORN axons passed. The ORNs are continually regenerated from the basal cells, progenitor cells of ORNs. The normal ORNs prevent the intracranial invasion of *S. pneumoniae*, allowing only a small amount of *S. pneumoniae* intrusion. TRPV1 on ORNs indirectly blocks the intracranial invasion by promoting ORN regeneration or suppressing ORN damage. Moderate induction of mediators such as IL-6 and NFκB via TRPV1 activation in the trigeminal nerve can eliminate a small number of bacteria in the OB. Under ORN damage, large amounts of *S. pneumoniae* can invade the cranium. An excessive trigeminal TRPV1-mediated response results in the activation of STAT3 together with IL-6. The co-activation of NFκB and STAT3 triggers IL-6 amplification, leading to cytokine storms with elevated TNF-α, which can be lethal.

IL-6 acts as a facilitator of other inflammatory cytokine/chemokine amplification (50). TRPV1 stimulation induces IL-6 amplification and exacerbates encephalomyelitis with excessive induction of other pro-inflammatory cytokines (51). This IL-6 amplification is driven by the co-activation of NFκB and STAT3, which triggers cytokine storms (52). In this study, increased intracranial invasion combined with intact trigeminal TRPV1 led to increased lethality. Massive intracranial invasion activated STAT3 along with NFκB via trigeminal TRPV1, and the induced IL-6 amplification could have triggered TNF-α production and lethality under OE damage (Fig. 9). The *cgrp* elevation in OB could be an indicator of TRPV1 activation in response to intracranial invasion. The *Cxcl1* levels in the OB were parallel to bacterial invasion but not to lethality. The relationship between pneumococcal meningitis and neutrophil induction was not clear in this model. Inflammatory cells and cytokine/chemokine induction peaked at 6 hours post-infection in a similar mouse model (53). The local inflammatory response in the nasal cavity might be above peak at 48 hours post-infection, resulting in no difference. TRPV1 was present on macrophages within the OE and OB (Fig. S3). Macrophage infiltration into the CNS is thought to be more prominent in hematogenous infection, accompanied by systemic inflammation and blood-brain barrier disruption (54). It was difficult to directly compare CNS involvement across different infection routes, which will underlie why statistical significance was not observed. The role of TRPV1 in inflammatory cells could not be determined in this study. Taken together, TRPV1 on the olfactory nerve inhibited CNS infection via maintenance of the OE barrier, whereas TRPV1 on the trigeminal nerve facilitated inflammation of CNS infection via induction of IL-6 and TNF-α. Steroids are used for an effective treatment for meningitis, because they may inhibit the trigeminal TRPV1-mediated inflammatory response.

The next question was whether TRPV1 stimulation of the nasal cavity could suppress CNS infection. Inhalation of TRPV1 stimulants has been clinically applied to prevent aspiration pneumonia in elderly people by inducing the pharyngeal reflex (55). Intranasal administration is a common way of olfactory rehabilitation methods for olfactory disorders, so it can be applied to TRPV1 stimulation (56). Methimazole induces ORN apoptosis (44). Pre-administration of capsaicin inhibited cisplatin-induced skeletal muscle atrophy by downregulating apoptosis via activation of TRPV1 (57). Rodent ORNs take approximately 1 month to regenerate (58). Therefore, the capsaicin-pretreated model in this study inhibited methimazole-induced ORN damage by a similar mechanism rather than by promoting ORN regeneration. The histological and olfactory behavioral test supported this hypothesis. Intranasal administration of TRPV1 stimulant appeared to have a predominant effect on the olfactory nerve and acted against non-haematogenic CNS infections.

There is one limitation to this study. Because RTX treatment can affect nociceptive nerves throughout the body, it cannot be ruled out that TRPV1 outside the trigeminal nerve may have influenced the outcomes. However, this study investigated local nasal responses without bloodstream infection. The impact was considered to be limited.

In summary, we investigated the role of TRPV1 on the olfactory nerve and the trigeminal nerve in intracranial invasion after intranasal infection with *S. pneumoniae*. TRPV1 on the olfactory nerve protected against intracranial invasion, whereas TRPV1 on the trigeminal nerve could increase lethality due to excessive inflammatory responses. Intranasal TRPV1 stimulation was superior in inhibiting intracranial invasion and could control lethal CNS infection. These findings suggest a novel strategy for infection prevention targeting TRPV1.

## MATERIALS AND METHODS

### Mice

Six-week-old mice were used in the studies. Wild-type (WT) C57BL/6J mice were purchased from Charles River Laboratories, Japan, and TRPV1 knockout (KO) mice (JAX 003770: B6.129X1-Trpv1^tm1Jul/J^) of the same genetic background were obtained from the Jackson Laboratory. The TRPV1 KO mice lack the *trpv1* gene by homologous recombination in embryonic stem cells (59). All mice were kept in a conventional animal facility at Wakayama Medical University.

### Methimazole and resiniferatoxin pre-treatment for TRPV1 ablation model

To ablate TRPV1 on the olfactory nerve, methimazole (75 mg/kg; FUJIFILM Wako Pure Chemicals Co.) dissolved in 0.9% saline was intraperitoneally administered 2 days before *S. pneumoniae* infection. Methimazole selectively damages ORNs, including TRPV1, with damage peaking 24 hours to 7 days after administration in a murine model (43, 60). Resiniferatoxin was used to ablate TRPV1 on the trigeminal nerve. RTX was administered intraperitoneally to mice 30 days before *S. pneumoniae* infection at doses of 80, 100, and 150 ng/body weight (g) over three consecutive days (61). Ablation by RTX was confirmed by injecting mice with 50 µg capsaicin in the cheek, followed by wiping less than 20 times within 5 minutes (61).

### Bacterial strains and growth conditions

Three strains of *S. pneumoniae* were used. Serotype 6A, P2431, was kindly transferred from J.N. Weiser of New York University. 6A is one of the most virulent encapsulated strains, which can induce sepsis after intranasal challenge (25). Serotype 19F, EF3030, was used as another encapsulated strain. A non-encapsulated *S. pneumoniae*, MNZ11, which was kindly provided by M.H. Nahm of the University of Alabama at Birmingham, is a clinical isolate (26). 6A and 19F can also cause meningitis in humans. These two strains are covered by PCV13, a capsular polysaccharide vaccine (27). A non-vaccine strain, MNZ11, is known to have the ability to colonize in the nasal cavity and cause invasive infection (26, 53, 62). All bacterial strains were cultured in tryptic soy broth (TSB; Becton Dickinson, Franklin Lakes, NJ, USA) until they reached the mid-exponential phase at 37°C, achieving an optical density at 600 nm (OD_600_) of 0.5. For infection, bacteria were washed and diluted in sterile phosphate-buffered saline (PBS) to the desired OD_600_.

For quantitative bacterial analysis, triplicate 10-fold serial dilutions were plated on blood sheep agar plates. The plates were then incubated overnight at 37°C with 5% CO_2_. Bacterial strains were stocked in 20% glycerol at −80°C until needed.

### Quantitative analysis of *S. pneumoniae* in tissues

After intranasal infection, mice were euthanized with isoflurane, and we collected nasal lavage, blood, OB, spleen, and BALF (29). Nasal lavage was obtained from the nose by intratracheal injection of 200 µL PBS. The OB and spleen were washed with sterile PBS and suspended in 1 mL PBS. Supernatants were collected after brief centrifugation. BALF was collected by flushing the lung four times with 800 µL PBS (53). We aseptically collected 20 µL of each liquid before serial dilution on blood sheep agar. After overnight incubation at 37°C with 5% CO_2_, we counted the *S. pneumoniae* colonies. The detection limit was 666 CFU/mL. To determine the dynamics of *S. pneumoniae* in MZ-mice, we also collected nasal lavage, blood, and OB at 1, 6, and 24 hours post-infection.

For intracranial infection, mice were inoculated with 7,000 CFUs of 6A suspended in 10 µL PBS via a 31G needle inserted into the frontal skull after a 1 cm skin incision. The skin was closed with 5–0 nylon sutures after infection. Survival was monitored, and 20 µL of blood was collected from surviving mice via the retro-orbital sinus for bacterial count every 24 hours after infection.

### Cytokine quantification by ELISA

Nasal lavage and OB were collected at 48 hours post-infection. Each OB was homogenized in 1 mL PBS and centrifuged at 500 × *g* for 5 minutes to obtain supernatants. The concentrations of IL-6 and TNF-α were measured as pg/mg from standard curves using ELISA kits (Proteintech, Rosemont, IL, USA) according to the manufacturer’s instructions.

### mRNA assay using quantitative real-time PCR

Nasal mucosa was rinsed intratracheally with 600 µL of RNA Lysis Buffer (Qiagen) containing 10% 2-mercaptoethanol. Similarly, OBs were collected and suspended in 600 µL of RNA lysis buffer. The lysate contained intracellular mRNA from OE and OB. The mRNA was purified using the RNeasy Mini Kit (Qiagen) with RNase-Free DNase I and DNase 10 Reaction Buffer (Promega). The RNA was then reverse transcribed into complementary DNA (cDNA) using the High-Capacity cDNA Reverse Transcription Kit (Applied Biosystems). Quantitative real-time PCR was performed with PowerUP SYBR Green Master Mix (Applied Biosystems). For each PCR reaction, 10 ng of cDNA was used as the template, combined with 0.5 mM forward and reverse primers. Reaction validation was achieved by amplification of glyceraldehyde-3-phosphate dehydrogenase. For each sample, the assay was performed in duplicate using two wells per gene, and the average value was used. The comparative delta threshold cycle (∆∆CT) method was applied. Primer sequences are shown in Table 1.

**TABLE 1:** Primers

Primers	Sequences in use
GAPDH-F	5′-AGGTCGGTGTGAACGGATTTG-3′
GAPDH-R	5′-TGTAGACCATGTAGTTGAGGTCA-3′
CXCL1-F	5′-CTGGGATTCACCTCAAGAACATC-3′
CXCL1-R	5′-CAGGGTCAAGGCAAGCCTC-3′
CGRP-F	5′-GGACTTGGAGACAAACCACCA-3′
CGRP-R	5′-GAGAGCAACCAGAGAGGAACTACA-3′
IL-6-F	5′-AGTTGCCTTCTTGGGACTGA-3′
IL-6-R	5′-TCCACGATTTCCCAGAGAAC-3′
TNFα-F	5′-TGTGCCTCAGCCTCTTCTC-3′
TNFα-R	5′-GAGCCCATTTGGGAACTTCT-3′
NGF-F	5' -GTTTTGCCAAGGACGCAGCTTTC-3′
NGF-R	5' -GTTCTGCCTGTACGCCGATCAA-3′

### Determination of inflammatory cell induction using flow cytometry

We applied 200 µL of nasal lavage or OB suspension to a 96-well plate, centrifuged it at 1,500 rpm for 2 minutes, and then drained the supernatant. The pellets were resuspended in PBS containing 1% bovine serum albumin. All subsequent steps were carried out on ice at 4°C. As a control, 100 µL of the bone marrow from the mouse was used. The cells were incubated for 30 minutes at 4°C in the dark with 25 µL of a 1:150 dilution of antibodies: anti-CD11b-V450, anti-F4/80-phycoerythrin (PE) (BioLegend, San Diego, CA, USA), anti-Ly6G-peridinin chlorophyll protein (PerCP)-Cy5.5, and anti-CD45-allophycocyanin (APC)-Cy7 (BD Biosciences). Fc receptors were blocked with a 1:200 dilution of anti-CD16/32 (BioLegend) for 15 minutes. Cells were then fixed with 4% paraformaldehyde. Flow cytometric analysis was performed by FACSVerse (BD). Neutrophils were identified by the presence of CD11b+, Ly-6G+, and CD45+ markers, whereas macrophages were characterized by CD11b+, Ly-6G−, and F4/80+ markers (29).

### Capsaicin pre-treatment

To investigate the effects of TRPV1 stimulation in the nasal cavity, 10 µg/g capsaicin was administered intranasally daily for one week prior to 75 mg/kg methimazole treatment. The day of *S. pneumoniae* infection was set as day 0. Mice were euthanized, and nasal lavage, blood, and OB were collected to quantify bacterial counts. Mice heads were collected for the histological examinations described below.

### Histologic examination

The heads of euthanized mice were immersed in 4% paraformaldehyde for 48 hours. The samples were then decalcified and dehydrogenated before paraffin embedding. Coronal sections of the nasal cavity were prepared from paraffin blocks at a thickness of 5 µm. Sections containing the OB in the center of the eyes were used for HE staining and the immunostaining as described below.

### Immunohistochemistry and immunofluorescence staining

The primary antibodies diluted in PBS and used in this study were as follows. For Fig. 1 and Fig. S1: anti-olfactory marker protein (OMP; goat polyclonal, 1:10,000 dilution; FUJIFILM Wako), anti-TRPV1 antibody (mouse monoclonal, 1:300 dilution; Abcam), anti-substance P antibody (rabbit polyclonal, 1:400 dilution; Abcam). For Fig. 5: anti-olfactory marker protein (OMP; goat polyclonal, 1:16,000 dilution; FUJIFILM Wako), anti-*S*. *pneumoniae* serotype 6A capsular antibody (rabbit monoclonal, 1:2,000 dilution; Denka), anti-claudin-1 antibody (rabbit polyclonal, 1:400 dilution; abcam). For Fig. 7: anti-olfactory marker protein (OMP; goat polyclonal, 1:16,000 dilution; FUJIFILM Wako), NF-κB p65 antibody (rabbit monoclonal, 1:2,000 dilution; Cell Signaling), Phospho-Stat3 (pSTAT3; rabbit monoclonal, 1:75 dilution; Cell Signaling). For Fig. S3: anti-TRPV1 antibody (mouse monoclonal, 1:300 dilution; Abcam), anti-Iba1 antibody (rabbit monoclonal, 1:5,000 dilution; Abcam). Immunofluorescence staining was performed by Morpho Technology. Images were captured using NDP. View2 (HAMAMATSU).

### Histologic analysis

The OE on the nasal septum was assessed under ×400 magnification. The thickness of OE was measured at three distinct levels on both the left and right sides of the coronal section, resulting in a total of six measured regions. The levels were 250, 500, and 750 µm from the upper end of the nasal cavity (63, 64). The proportion of OMP-positive cells was determined by counting positive and negative cells within 100 µm of OE.

### Buried food test

Mice were subjected to a first test 24 hours after the start of fasting and a subsequent second test after a further 24 hours of continuous fasting. Thus, the study consists of fasting for a total of 48 hours and two tests (65). On the test day, the mice were transferred to a new cage and provided with a single piece of food 10 minutes before the start. This step, referred to as “initiation,” is crucial for helping the mice to recall the food scent. A single food pellet was buried 1 cm beneath the bedding, which was approximately 5 cm deep, in one corner of the cage (38 × 24 × 20 cm). Each mouse was tested individually. Latency was defined as the time between when the mouse was released into the center of the cage and when it found the buried pellet. The test duration was 300 seconds. If mice could not find the pellet, it was recorded for 300 seconds.

### Statistical analyses

All statistical analyses were performed by GraphPad Prism 10.0 (GraphPad Software Inc.). Differences were determined by the Mann-Whitney U test or Fisher’s exact test (comparison of two groups), or the Kruskal-Wallis test with Dunn post hoc analysis (comparison of multiple groups). A *P* value of <0.05 was considered to be statistically significant.

## Data Availability

All data sets used and/or analyzed during the current study are included in the figures and supplemental figures and are available from the corresponding author on reasonable request.

## SUPPLEMENTAL MATERIAL

Fig. S1 mbio.01335-25-s0001.pdf TRPV1 distribution in nasal mucosa following MZ or RTX treatment.

Fig. S2 mbio.01335-25-s0002.pdf Inflammatory responses in the nasal cavity and olfactory bulb via quantitative real-time PCR and flow cytometry.

Fig. S3 mbio.01335-25-s0003.pdf TRPV1 presence on macrophages in olfactory epithelium and olfactory bulb.

ASM does not own the copyrights to Supplemental Material that may be linked to, or accessed through, an article. The authors have granted ASM a non-exclusive, world-wide license to publish the Supplemental Material files. Please contact the corresponding author directly for reuse.
