# Supplementary material for: Trigeminal TRPV1 regulates pneumococcal nose-to-brain invasion via IL-6/TNF-α signals
Source: mBio. 2025 Aug 18;16(9):e01335-25. doi: 10.1128/mbio.01335-25 (PMC12421820; doi:10.1128/mbio.01335-25)
Supplement: Fig. S1 — TRPV1 distribution in nasal mucosa following MZ or RTX treatment. [file mbio.01335-25-s0001.pdf]

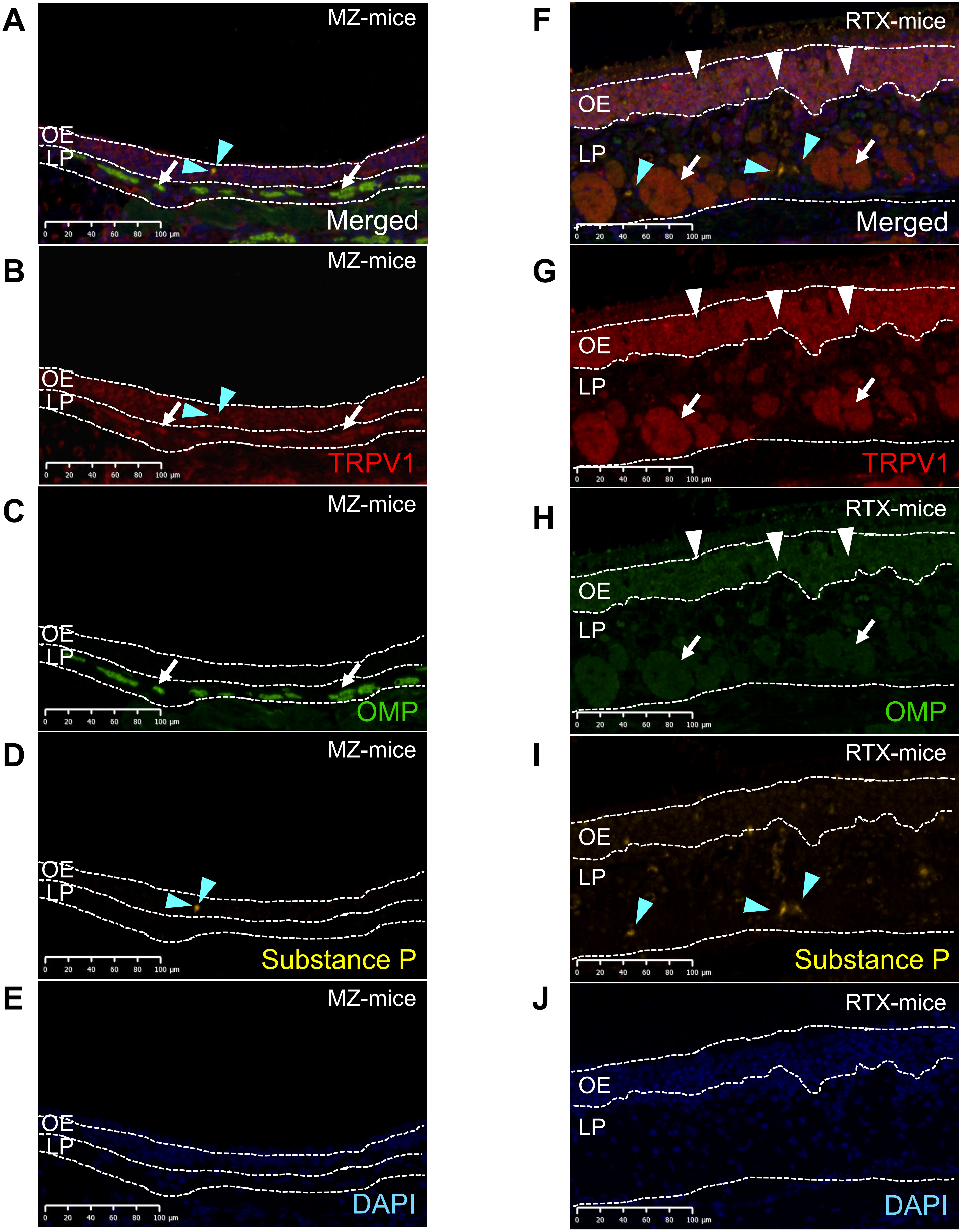

Figure S1. TRPV1 distribution in nasal mucosa following MZ or RTX treatment. TRPV1 distribution in the nasal mucosa was evaluated by immunofluorescent staining following MZ (A–E) or RTX (F–J) treatment. A, F: Merged; B, G: TRPV1 (red); C, H: OMP (green); D, I: Substance P (yellow); E, J: DAPI (blue). White arrowheads, white arrows, and blue arrowheads represent ORN cell bodies, olfactory nerve fascicles, and trigeminal nerve fibers, respectively. Scale bar shown bottom right. LP, lamina propria; OE, olfactory epithelium; TRPV1, transient receptor potential vanilloid 1; OMP, olfactory marker protein; MZ, methimazole; RTX, resiniferatoxin; DAPI, 4',6-diamidino-2-phenylindole.
