# Supplementary material for: Trigeminal TRPV1 regulates pneumococcal nose-to-brain invasion via IL-6/TNF-α signals
Source: mBio. 2025 Aug 18;16(9):e01335-25. doi: 10.1128/mbio.01335-25 (PMC12421820; doi:10.1128/mbio.01335-25)
Supplement: Fig. S2 — Inflammatory responses in the nasal cavity and olfactory bulb via quantitative real-time PCR and flow cytometry. [file mbio.01335-25-s0002.pdf]

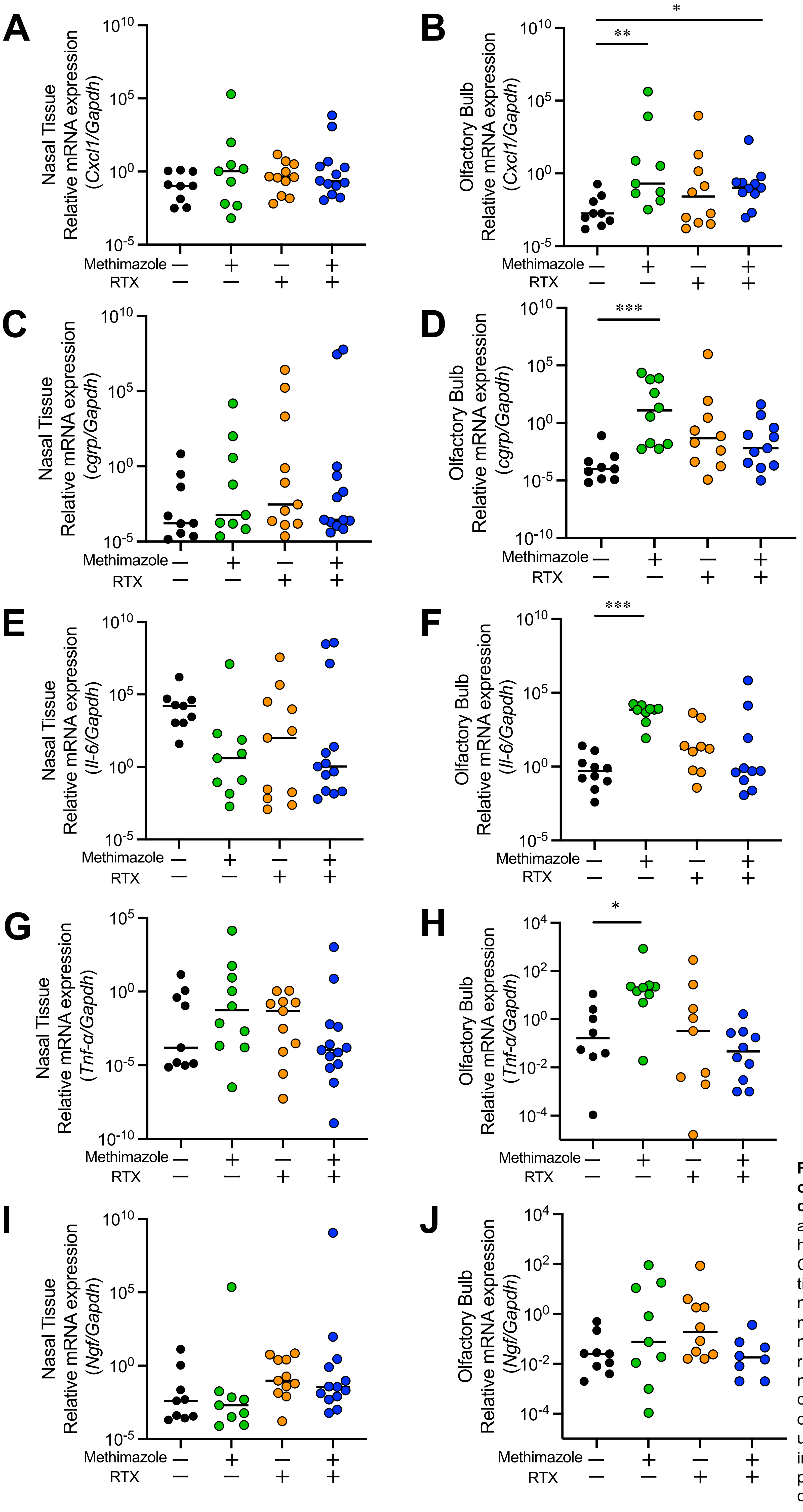

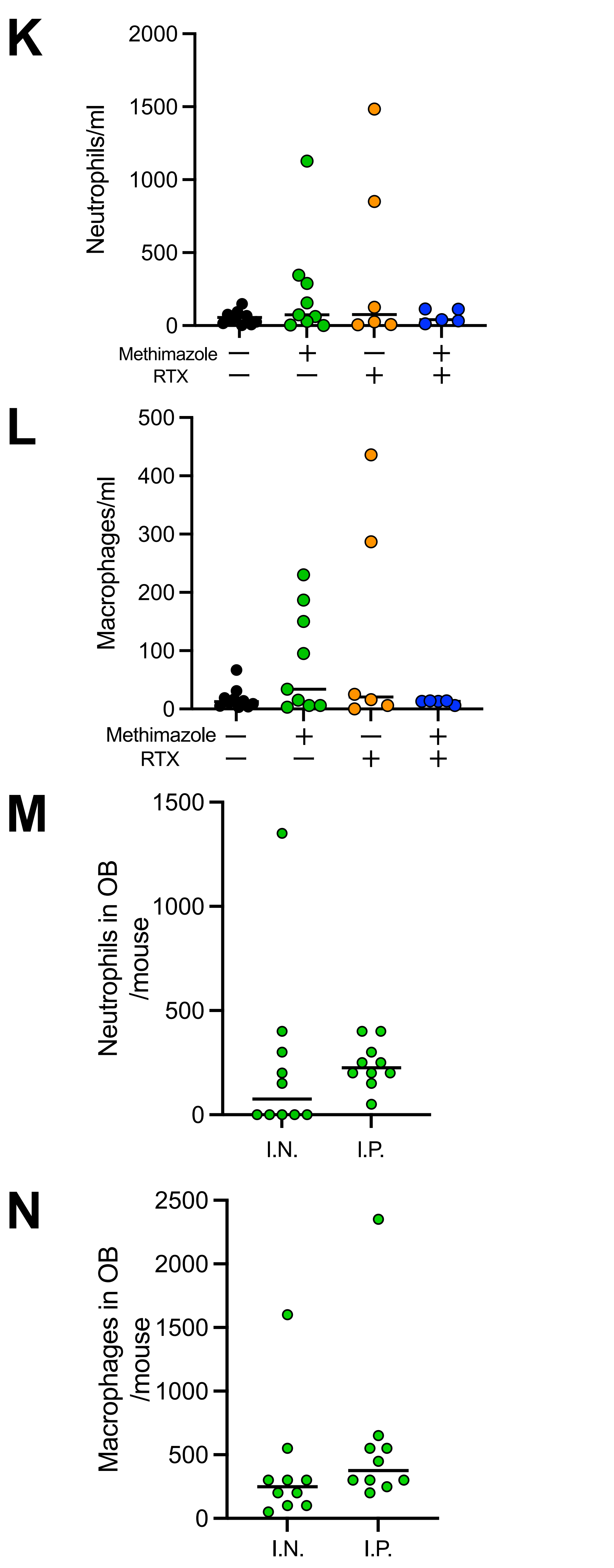

Figure S2. Inflammatory responses in the nasal cavity and olfactory bulb via quantitative real-time PCR and flow cytometry. WT mice were pretreated with MZ and/or RTX according to the procedures shown in Fig. 2A, and euthanized at 48 hours post-infection with 6A at 7000 CFUs/mouse. A-J. Cytokines/chemokines, neuropeptides and growth factors in nasal tissue (A, C, E, G, I) and olfactory bulb (B, D, F, H, J). K. The number of neutrophils in nasal lavage. L. The number of macrophages in nasal lavage. Black dots represent untreated mice. n=8-10. Green dots represent MZ-mice. n=9-11. Orange dots represent RTX-mice. n=9-11. Blue dots represent MZ-RTX-mice. n=8-13. M, N. The number of neutrophils (M) and macrophages (N) counts in OB suspension at 48 hours after intranasal (7000 CFUs) or intraperitoneal (100 CFUs) infection of MZ-pretreated WT mice, using equivalent survival doses. n=10. I.N., intranasal. I.P., intraperitoneal. Statistical comparisons with the control were performed using Kruskal-Wallis test with Dunn's multiple-comparison test. * p<0.05, ** p<0.01 and *** p<0.005.
