# Supplementary material for: Trigeminal TRPV1 regulates pneumococcal nose-to-brain invasion via IL-6/TNF-α signals
Source: mBio. 2025 Aug 18;16(9):e01335-25. doi: 10.1128/mbio.01335-25 (PMC12421820; doi:10.1128/mbio.01335-25)
Supplement: Fig. S3 — TRPV1 presence on macrophages in olfactory epithelium and olfactory bulb. [file mbio.01335-25-s0003.pdf]

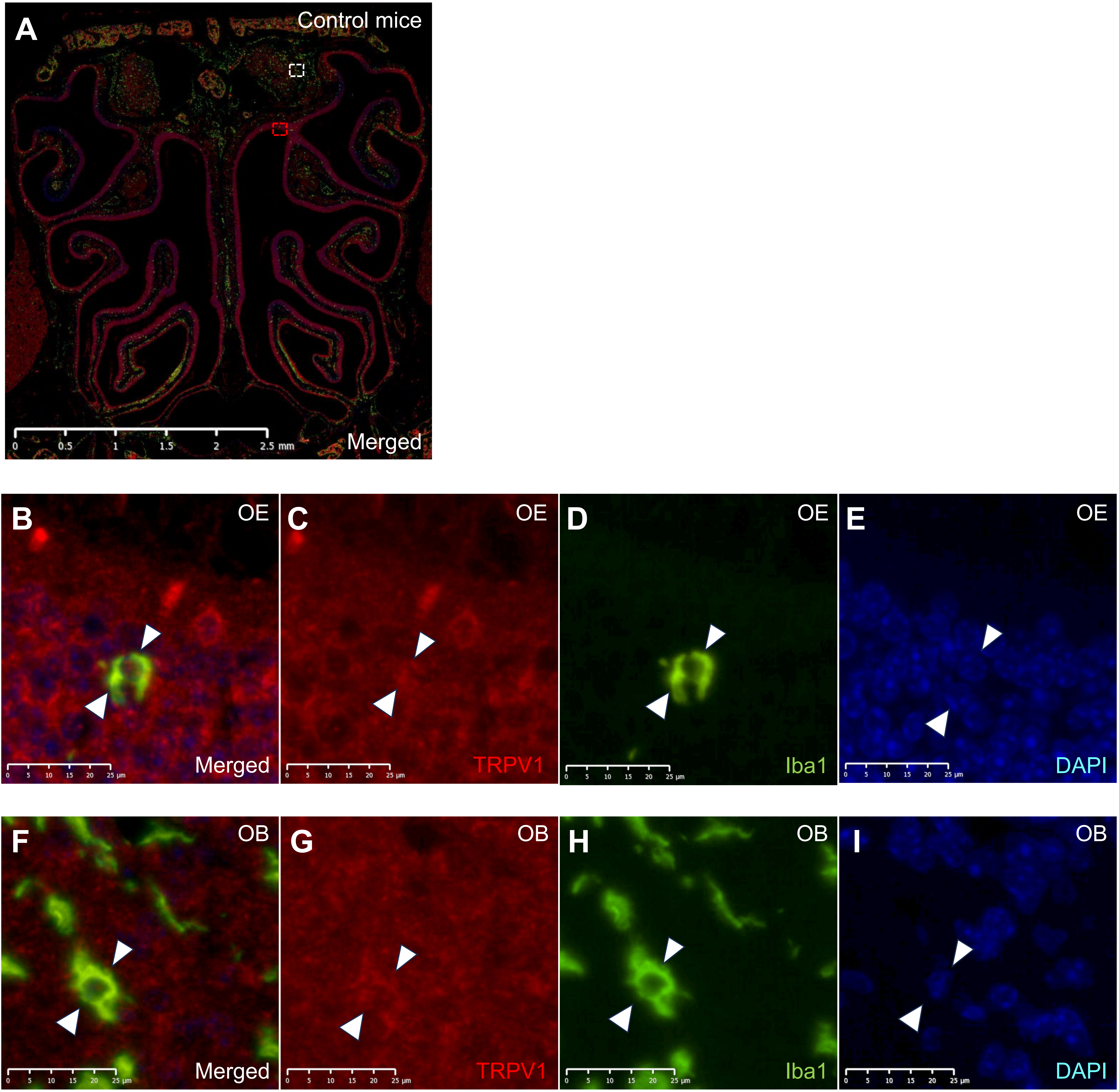


**Figure S3. TRPV1 presence on macrophages in olfactory epithelium and olfactory bulb.** The immunofluorescence images of coronal sections from the representative control mouse in Fig. 4 – 7, using anti-TRPV1 and anti-Iba1 antibodies. A. Low magnification image including OE and OB. The areas outlined by red dashed lines and white dashed lines represent OE and OB, respectively. B–E. High magnification images of the red boxed area in A. F–I. High magnification images of the white boxed area in A. B, F: Merged. C, G: TRPV1(red). D, H: Iba1(green). E, I. DAPI (blue) . White arrowheads indicate TRPV1-positive macrophages. A scale bar is shown in the lower right corner of each image. OE, olfactory epithelium; OB, olfactory bulb; TRPV1, transient receptor potential vanilloid 1.
